# A Novel Strain of *Bacillus cereus* with a Strong Antagonistic Effect Specific to *Sclerotinia* and Its Genomic and Transcriptomic Analysis

**DOI:** 10.3390/microorganisms12030611

**Published:** 2024-03-19

**Authors:** Wanfu Ma, Jinhao Ding, Qingyun Jia, Qianru Li, Shanhai Jiao, Xupeng Guo, Chengming Fan, Yuhong Chen, Zanmin Hu

**Affiliations:** 1 College of Tropical Agriculture and Forestry, Hainan University, Haikou 570228, China; mawanfu1234@163.com; 2 Key Laboratory of Seed Innovation, Institute of Genetics and Developmental Biology, Innovation Academy for Seed Design, Chinese Academy of Sciences, Beijing 100101, China; jhding@genetics.ac.cn (J.D.); qyjia@genetics.ac.cn (Q.J.); qrli@genetics.ac.cn (Q.L.); guoxvpeng@163.com (X.G.); zmhu@genetics.ac.cn (Z.H.); 3 College of Advanced Agricultural Sciences, University of Chinese Academy of Sciences, Beijing 100049, China; 4 AUSCA Oils and Grains Industries Co., Ltd., Fangchenggang 538000, China; jsh123456@126.com

**Keywords:** *Sclerotinia sclerotiorum*, *Bacillus cereus*, biocontrol, oilseed rape, antagonistic effect, multiomics analysis

## Abstract

Sclerotinia, which is caused by *Sclerotinia sclerotiorum*, is a severe disease of oilseed rape, which is an important oil crop worldwide. In this study, we isolated a novel strain of *Bacillus cereus*, named *B. cereus* HF10, from the rhizosphere soil of the reed on the seaside of Yagzhou Bay, Sanya city, Hainan Province, China. HF10 exhibited a significant antagonistic effect on *Sclerotinia sclerotiorum*, with an inhibition rate of 79%, and to other species in *Sclerotinia*, but no antagonistic effect was found on various other fungi or bacteria. HF10 had an 82.3% inhibitory effect on the *S. sclerotiorum* infection of oilseed rape leaves and a 71.7% control effect on Sclerotinia infection in oilseed rape based on in vitro and in vivo experiments, respectively. The genomics and transcriptomics of HF10 and its loss of the antifungal function mutant Y11 were analyzed, and the results provided insight into potential antifungal substances. Our work provides a novel strain, HF10, for developing a promising biological control agent against *Sclerotinia*, which infects oilseed rape and other plants.

## 1. Introduction

Oilseed rape is one of the major oil crops in the world with an oil content of 33–50%, and it is widely used for edible oil, feed, food, medicine, and industrial raw materials such as biodiesel [1]. It is cultivated mainly in China, Canada, Europe, India, and other regions [2]. China is the largest country for oilseed rape cultivation and canola oil consumption [3]. However, the production of oilseed rape plants has been severely affected by many kinds of diseases, including the diseases caused by viruses, bacteria, and fungi such as black shank disease and sclerotinia, which are fungus-derived diseases [4].

For fungal diseases, *Sclerotinia* pathogens are an important group of fungi with 90 species and a wide host range. It can infect at least 75 families, 278 genera, and 600 species of plants [5], including a variety of important economic crops belonging to the family Cruciferae, Solanaceae, Asteraceae, and Leguminosae [6,7].

In the genus *Sclerotinia*, *S. sclerotiorum*—which is a soilborne and airborne fungal pathogen—could cause severe sclerotium disease in oilseed rape in China, as well as in some other regions of the country [2]. It spreads quickly and has a stubborn vitality, and it can lead to the decay of oilseed rape leaves and stems, thereby resulting in a reduction in yield of up to 80% [8] and affecting the quality of oilseed rape [8].

Due to its various transmission modes, it is extremely difficult to control *S. sclerotiorum*. At present, the prevention and control of *Sclerotinia* in oilseed rape mainly involve the use of chemical agents, such as thiophanate-methyl, carbendazim, and fluopyram [9]; however, the long-term use of chemical agents not only affects the quality of oilseed rape-based products, but can also be detrimental to human health and the safety of the natural environment [10,11]. In addition, *Sclerotinia* strains resistant to chemical agents can be easily produced, thus resulting in increased chemical use [12].

Biological control is currently an important area of research in plant disease control. Compared with chemical control, biological control has the advantages of less environmental pollution and better biosafety [13,14,15,16,17]. In recent years, biocontrol agents have been widely used for plant disease control; for example, *Agrobacterium*, *Arthrobacter*, *Bacillus*, *Pseudomonas*, *Rhizobium*, *Serratia*, *Stenotrophomonas*, *Streptomyces*, and *Xanthomonas* have been described as responding with strong activity against fungal and bacterial pathogens [18]. The *Bacillus* species has been the most exploited beneficial bacteria for use as biopesticides. For sclerotium control, the agents developed from *Bacillus subtilis* and *Paenibacilus polymyxa* should not only effectively control crop sclerotium, but also significantly reduce the number of pathogenic fungi in plant rhizosphere soil and recruit additional beneficial microorganisms [19,20,21,22,23].

For antifungal bacteria, different species and even different strains from the same species have different antifungal effects due to the different effective metabolites produced by them, thereby resulting in significantly varied antifungal spectra and activities against the same pathogen [24]. In addition, most antifungal bacteria are antagonistic not only to fungal pathogens, but also to probiotic fungi, such as *Trichoderma* [25]. Therefore, it would be ideal to find antagonistic bacteria that are effective at treating certain plant diseases.

A new strain of *Bacillus cereus*, HF10, was identified in the present study, which responds specifically with strong antagonistic activity against the genus *Sclerotinia* but not against other types of filamentous fungi, including *Fusarium*, *Aspergillus*, *Trichoderma*, etc. Furthermore, this strain could significantly prevent *S. sclerotiorum* infection in oilseed rape. In addition, the genetic basis (the genome and transcriptome) of this strain was investigated. Our study is highly important for developing effective biological control agents for the environmentally friendly prevention of *Sclerotinia* in oilseed rape.

## 2. Materials and Methods

### 2.1. Materials

#### 2.1.1. Soil Samples

Soil samples were obtained from the rhizosphere soil of *Phragmites australis*, which were located along the shoreline of Yazhou Bay, Sanya city, Hainan Province, China. By excavating, soil samples were obtained from a depth of 10 cm below the surface, which was characterized as lateritic soil.

#### 2.1.2. Fungus Strains and Oilseed Rape Variety

The fungal strains used for antagonistic bacterial activity tests in this study were *S. sclerotiorum* WH13 (kindly provided by Professor Shenyi Liu, Institute of Oil Crops, Chinese Academy of Agricultural Sciences, Wuhan, China); *S. sclerotiorum* 1980, *S. sclerotiorum* P134, *S. sclerotiorum* Ep-1pn A367, *S. minor*, and *S. sclerotiorum* H1 (kindly provided by Professor Daohong Jiang, Huazhong Agricultural University, Wuhan, China); and *Aspergillus flavus*, *Fusarium equiseti*, *Trichoderma asperellum*, *Fusarium oxysporum*, *Fusarium graminearum*, *Fusarium verticillioides*, *Corynespora cassiicola*, *Colletotrichum horii*, *Alternaria gossypina*, *Cladosporium cladosporioides*, *Glomerella ciningulaia*, and *Mucor irregularis* (kindly provided by Professor Long Wang, Institute of Microbiology, Chinese Academy of Sciences, Beijing, China). *Bacillus subtilis* H01 and H02 were kept at our laboratory and were used for potential antifungal substance tests as the positive control.

Oilseed rape *Brassica napus* var. Westar was used for the *S. sclerotiorum* infection test.

#### 2.1.3. Culture Medium

Potato agarose medium (PDA medium, 200 g/L of potato, 20 g/L of glucose, 16 g/L of agar 15–, and pH = 7) was used for the fungal culture. LB liquid medium (5 g/L of yeast extract, 10 g/L of NaCl, and 10 g/L of tryptone, pH = 7) and LB solid medium (LB liquid medium with 15 g of agar powder) were used for the bacterial culture.

### 2.2. Isolation and Screening of Bacteria Antagonizing Fungal Pathogens

A 5 g dry soil sample was mixed with 45 mL of sterilized water using glass beads. The mixture was agitated for 30 min at 28 °C and 200 rpm. Subsequently, 1 mL of the solution was used for gradient dilution, and two dilutions, 10^−5^ and 10^−6^, were generated. The diluted solution was spread on LB solid media and cultured for 24 h. The colonies were identified, picked, and put into 1.5 mL centrifuge tubes with LB liquid media for subsequent screening, each with a unique number.

The plate confrontation method was used to screen for strains that antagonize the fungal pathogen *S. sclerotiorum* using the protocol described by described by Li et al. [26]. A colony with a high inhibition rate was selected for strain purification and further characterization.

The inhibition rate of the sterile fermentation supernatant of the selected bacteria against *S. sclerotiorum* was tested using the mycelial growth rate detection method described by Ma et al. [27]. Briefly, the selected bacteria were inoculated into triangular flasks with 100 mL of a LB medium and cultured for 24 h to 48 h at 28 °C and 200 rpm. The culture was then centrifuged at 10,000 rpm at 4 °C for 10 min, and the resulting supernatant was collected and filtered through a 0.22 μm filter membrane to eliminate bacteria, thereby yielding a sterile filtrate. The sterile fermentation filtrate was mixed with PDA media and diluted 10, 20, and 50 times. An 8 mm-long *S. sclerotiorum* cake was placed on the center of a Petri dish with a PDA medium mixed with a sterile fermentation supernatant. The inhibition rate was calculated for *S. sclerotiorum* in the control group, which was grown throughout the Petri dish. The experiment was repeated 3 times. The inhibition rate was calculated using the following formula: inhibition rate (%) = [(average diameter of colonies in the control group – average diameter of colonies in the treatment group)/average diameter of colonies in the control group] × 100%.

### 2.3. Characterization of the Selected Bacteria

#### 2.3.1. Physiological and Biochemical Characteristics of Strain HF10

The physiological and biochemical characteristics of the strain HF10, which had the highest inhibitory activity against *S. sclerotiorum*, were assessed through a battery of tests, including sugar fermentation, anaerobic growth, catalase, Voges–Proskauer (V-P), starch hydrolysis, gelatine liquefaction, oxidase, 7% sodium chloride growth, propionate, and nitrate reduction tests, using the methods described in a previous reference [28].

For the sugar alcohol fermentation test, a medium containing 1 g of (NH_4_)_2_HPO_4_, 0.2 g of MgSO_4_, 0.2 g of KCl, 0.2 g of yeast extract, and 1% sugar (alcohol) in a 1000 mL solution with a pH of 6.8~7.0 and 2 mL of 0.4% bromocresol purple solution, was used. The tested sugars (alcohols and glycosides) included glucose, sucrose, mannitol, lactose, galactose, maltose, and sorbitol. The HF10 strain was inoculated into the medium and cultured at 28 °C for 1, 3, and 5 days. The medium color was observed. Yellow indicated a positive result, whereas no color change indicated a negative result.

For the anaerobic test, HF10 was inoculated into the sealed tube with a LB liquid medium and cultured at 28 ± 1 °C for 2 to 4 days; then, the cell growth was visually compared with the control, which was without HF10.

For the catalase test, 3% hydrogen peroxide was added dropwise into the HF10 culture. Abundant gas bubble production indicated a positive result, otherwise a negative result was indicated.

For the Voges–Proskauer test, a medium containing 5 g of peptone, 5 g of glucose, and 5 g of NaCl in a 1000 mL solution with a pH of 7.0 ± 0.2 was used. The HF10 strain was inoculated into the medium and cultured at 28 ± 1 °C for 2 to 4 days. The culture broth and a 40% sodium hydroxide solution were mixed together in equal volume. Then, a small amount of creatine was added into the mixture. The medium color was observed. When the medium turned red within 10 min, it indicated a positive reaction.

For the starch hydrolysis test, a medium (starch agar medium) containing 5 g of peptone, 5 g of NaCl, 5 g of soluble starch, and 20 g of agar in a 1000 mL solution with a pH of 7.2 was used. The HF10 strain was inoculated onto starch agar plates and cultured at 28 ± 1 °C for 24~48 h. Then, an iodine reagent was dropped onto the surface of the culture plate. The medium color was immediately observed.

For the gelatine liquefaction test, a medium containing 5 g of peptone and 120 g of gelatin in a 1000 mL solution with a pH of 7.2~7.4 was used. The amount of the HF10 strain was inoculated by puncturing into the gelatin layer to an approximately 2/3 depth (a total of 45 cm in a tube) and cultured at 20~22 °C for 3~4 days. When liquefaction was observed, it indicated a positive result, otherwise a negative result was indicated.

For the oxidase test, 1~2 drops of reagents (Kovacs’ reagent or an improved Ewing’s reagent) were applied onto the slant culture of an LB agar medium with HF10. A positive reaction was indicated by Kovacs’ reagent turning from pink to a deep purple, while the improved Ewing’s reagent turned blue. A negative reaction was indicated by the color not changing. The results were determined within a few minutes.

For the 7% NaCl test, a medium containing 5 g of yeast extract, 10 g of peptone, and 70 g of NaCl in a 1000 mL solution with a pH of 7.0 ± 0.2 was used. The HF10 strain was inoculated into the medium and cultured at 28 ± 1 °C for 2 to 4 days; then, the growth of the HF10 strain was visually compared with the control, which did not contain HF10.

For the sodium propionate test, a medium containing 1 g of diammonium hydrogen phosphate, 1 g of potassium dihydrogen phosphate, 0.2 g of MgSO_4_·7H_2_O, 5 g of NaCl, and 2 g of sodium propionate in a 1000 mL solution with a pH of 7.0 ± 0.2 and 10 mL of a 1% bromothymol blue aqueous solution was used. HF10 was inoculated into the medium and cultured at 28 ± 1 °C for 48 h. Using the culture medium without an inoculation of the HF10 strain was utilized as a control. A change in the medium color from green to blue indicated a positive result, while a lack of color change indicated a negative result.

For the nitrate reduction test, a medium containing 20 g of peptone, 5 g of NaCl, and 1 g of potassium nitrate in a 1000 mL solution with a pH of 7.0 ± 0.2 was used. The following reagents were prepared: a methyl solution (0.5 g of p-aminobenzenesulfonic acid and 150 mL of a 10% acetic acid solution), an ethyl solution (0.1 g of α-naphthylamine, 20 mL of distilled water, and 150 mL of a 10% acetic acid solution), and a diphenylamine reagent (0.5 g of diphenylamine, 20 mL of distilled water, and 100 mL of concentrated sulfuric acid). The HF10 strain was inoculated into the medium and cultured at 28 ± 1 °C for 2 to 4 days. One drop each of the methyl solution and ethyl solution was added into the culture, and the solution color changes were observed. If the solution turned red, orange, or brown, it indicated a positive nitrate reduction test. If no color changed, 1–2 drops of diphenylamine reagent was added and a negative nitrate reduction could be determined when the solution turned blue, otherwise a positive nitrate reduction was determined.

#### 2.3.2. 16S rDNA Sequencing and Phylogenetic Tree Construction of the HF10 Strain

16S rDNA was obtained by PCR using the universal primers 27F (5′-AGAGTTTGATCCTGGCTCAG-3′) and 1492R (5′-TACGGCTACCTTGTTACGACTT-3′), and it was sequenced by Sangon Bioengineering (Shanghai) Co., Ltd., Beijing Branch (Beijing, China). The size of the amplicon was 1542 bp. The PCR amplification system was composed of 1.5 μL of primer 27F and 1492R, 30 μL of PCR Mix, 24 μL of ddH_2_O, and 3 μL of DNA. The PCR amplification procedure was conducted as follows: pre-denaturation at 95 °C for 4 min, denaturation at 95 °C for 1 min, annealing at 55 °C for 1 min, extension at 72 °C for 2 min (for 30 cycles), final extension at 72 °C for 10 min, and storage at 4 °C. Based on the 16S rDNA sequence, a phylogenetic tree was constructed using MEGA-X (10.2.4) software.

### 2.4. Determination of the Antifungal Activity Spectrum of the HF10 Strain

The antagonistic activity of HF10 against pathogenic fungi was assessed using the plate confrontation culture method, in which the pathogenic fungi described in Section 2.1.2 were used as the targets. The experiment was repeated 3 times.

### 2.5. Test of the Effect of the HF10 Strain on the S. sclerotiorum Infection of Oilseed Rape In Vitro

The activated HF10 strain was inoculated into a 250 mL flask with 100 mL of an LB medium and cultured for 24 h at 30 °C, with agitation, and at 200 rpm. After fermentation, the mixture was centrifuged and the resulting suspension was diluted with distilled water to a concentration of 10^6^ cfu/mL.

Healthy oilseed rape leaves that exhibited a consistent development at the six leaf stage were selected. Specifically, the mature leaves of the oilseed rape that grow for 5 weeks. The leaf surface was sterilized with 75% alcohol for 15 s, washed with sterile water, and then air-dried naturally. A bacterial suspension of the HF10 strain at a concentration of 10^6^ cfu/mL was sprayed onto the surface of the oilseed rape leaves and allowed to air dry naturally in the shade. Subsequently, the activated *S. sclerotium* cake, which was 8 mm in diameter, was inoculated on the middle surface of the leaves. The leaves were then placed in a humidified box and incubated at 25 °C for 3 days. The experiment was conducted in triplicate, and 10 oilseed rape plants were used each time.

The effective rate was calculated using the following formula: effective rate = [(the infected area of control leaves – the infected area of treated leaves)/(the infected area of control leaves)] × 100%.

### 2.6. Effect of the HF10 Strain on the S. sclerotiorum Infection of Oilseed Rape Leaves In Vivo

When the oilseed rape plants had grown to the six leaf stage, a suspension of the HF10 Strain at a concentration of 10^6^ cfu/mL was evenly sprayed on the surface of the leaves, after which the plants were allowed to air dry naturally. After a 7-day incubation period, the larger leaves that were present at the consistent development stage were selected from the different plants. Activated *S. sclerotiorum* was prepared into an 8 mm diameter cake and inoculated onto the leaf surface with and without the spraying of the HF10 strain. The oilseed rape plants inoculated with *S. sclerotiorum* were covered with a transparent plastic cover to maintain air humidity, as well as to promote infection and disease development. The experiment was conducted in triplicate, and 10 oilseed rape plants were used each time.

The disease index was calculated after 7 days of *S. sclerotiorum* infection based on the classification standard of the oilseed rape sclerotia. The grading criteria for disease severity were as follows: Grade 0, no disease; Grade 1, the number of diseased leaf stalks on the oilseed rape plant was less than 1/3 and the length of the disease spot on the main stem was not more than 3 cm; Grade 2, 1/3 to 2/3 of the leaf stalks affected; or Grade 3, the number of diseased leaf stalks was less than 1/3 but the length of the disease spot on the main stem was more than 3 cm. With respect to Grade 3, the number of diseased leaf stalks on the oilseed rape plant was more than 2/3, or the number of diseased leaf stalks was less than 2/3, but the length of the disease spot on the middle and lower parts of the main stem was more than 3 cm.

Disease index = ∑(number of plants in each severity class × number of plants in each severity class)/(total number of investigated plants × 3) × 100

Control effect (%) = [(control group disease index − treated group disease index)/control disease index] × 100

### 2.7. Colonization Ability of the HF10 Strain on Oilseed Rape Leaves

The colonization ability of the HF10 strain on oilseed rape leaves was determined by a previously described method [29]. Briefly, the HF10 strain was cultured in an LB medium that was supplemented with 50 μg/mL of spectinomycin. The culture medium was removed by centrifuging 100 mL of the HF10 bacterial solution at a concentration of 6 × 10^8^ cfu/mL, diluting the mixture 10 times with distilled water, and then uniformly spraying the mixture on the surface of the oilseed rape leaves at the six leaf stage. Sterilized water containing 50 μg/mL of spectinomycin was sprayed on the plants in the control group, and the experiment was repeated 3 times, with 10 leaves measured each time. The treated leaves were collected to determine the colonization of the HF10 strain after 7 days using the following method.

The surface of the leaves was disinfected with 75% ethanol solution for 15 s, washed 3 times with sterile water, weighed and ground into a homogenate in a sterile mortar, dissolved in 10 mL of sterile water, kept for 30 min, and diluted 1000 times. The number of bacteria was determined by the plate gradient dilution method. A 100 μL aliquot of the above dilution was spread onto an LB solid medium, which was supplemented with 50 μg/mL of spectinomycin and cultured for 24 h at 37 °C. The number of colonies (cfu) per gram of fresh tissue was calculated. The experiment was repeated 3 times.

### 2.8. Investigation of the Antifungal Substances of the HF10 Strain

#### 2.8.1. Isolation and Detection of Antimicrobial Lipopeptides

The antimicrobial lipopeptides from the HF10 strain were extracted through acid precipitation [30]. In detail, upon completion of the fermentation on the HF10 strain LB media, 50 mL of fermentation broth was collected and centrifuged at 10,000 to 11,000 rpm for 10 min at 4 °C to separate the supernatant. The supernatant was then acidified by adding concentrated hydrochloric acid to adjust the pH to 2.0, which was followed by overnight refrigeration at 4 °C, and the precipitate was then obtained after centrifugation. The precipitate was air-dried and subsequently dissolved in 5 mL of HPLC-grade methanol, after which the mixture was filtered through a 0.22 μm sterile filter for the detection of lipopeptides via HPLC (Dalian Elite, China Analytical Instruments Co., Ltd., Dalian, China, Production of Agress1100). HPLC was carried out using an Agilent ZORBAX SB-C18 (3.0 × 150 mm) at 28 °C and a UV detector (at a wavelength of 214 nm). The loading volume was 20 µL. The flow rate was 1 mL/min. Mobile phase A: (0.1% TFA, trifluoroacetic acid) + water; B: HPLC grade acetonitrile + (0.1% TFA), elution gradient: 0 min: 70% A; 30% B; 5 min: 52% A; 48% B; 40 min: 44% A; 56% B; 45 min: 30% A; 70% B; 60 min: 0% A; and 100% B.

#### 2.8.2. Detection of Siderophores

The detection of siderophores was carried out using a previously reported method [31]. Single colonies of HF10 were carefully chosen and inoculated onto solid CAS plates [32]. The cultures were then incubated in a constant temperature incubator at 30 °C for 7 days, after which the presence of yellow halos around the colonies was observed. The *B. subtilis* H01 and *B. subtilis* H02 strains, which were screened in the laboratory, were used as the positive controls. The experiment was repeated 3 times.

#### 2.8.3. β-1,3 Glucanase Assay

The activated HF10 cells were inoculated into a glucanase detection medium containing 5 g of Wolfiporia cocos powder, 1 g of KH_2_PO_4_, 1 g of NaNO_3_, 0.5 g of KCl, 0.5 g of MgSO_4_·7H_2_O, 0.5 g of FeSO_4_·7H_2_O, 0.05 g of methylene blue, and 20 g of agar in a 1000 mL [33] solution, which was incubated at 30 °C for 72 h to observe the formation of hydrolysis circles. The *B. subtilis* H01 strain, which was screened in the laboratory, was used as the positive control. The experiment was repeated 3 times.

### 2.9. Multiomics Analysis of the HF10 Strain and Its Loss of the Antifunctional Function Mutant Y11

#### 2.9.1. Whole-Genome Sequence Analysis of HF10

The DNA of HF10 was extracted using a bacterial genome extraction kit (M5 Bacteria Genomic DNA Kit) from Beijing Jumei Biotechnology Co., Ltd., Beijing, China. The extracted DNA was subsequently submitted to Wuhan Huada Gene Technology Co., Ltd., Wuhan, China. for whole-genome sequencing and de novo assembly. The HF10 genome was sequenced using the PacBio Sequel II and DNBSEQ platforms at the Beijing Genomics Institute (BGI, Shenzhen, China). Zero-mode wavelet arrays of four SMRT cells were sequenced on the PacBio platform to generate the subread set. PacBio subreads (length < 1 kb) were removed. The program Canu was used for self-correction. Drafted genomic unitigs, which are uncontested groups of fragments, were assembled using the Canu high-quality corrected circular consensus sequence subread set. To improve the accuracy of the genome sequence information, GATK4 (https://www.broadinstitute.org/gatk/) was used to perform single-base corrections. Gene prediction was performed on the HF10 genome assembly by glimmer3 (http://www.cbcb.umd.edu/software/glimmer/) with hidden Markov models. tRNA, rRNA, and sRNA recognition was performed with tRNAscan-SE, RNAmmer, and the Rfam database, respectively. Tandem repeat annotations were obtained using Tandem Repeat Finder (http://tandem.bu.edu/trf/trf.html), and minisatellite DNA and microsatellite DNA were selected based on the number and length of repeat units. The Genomic Island Suite4 of Tools (GIST) was used for genomics land analysis (http://www5.esu.edu/cpsc/bioinfo/software/GIST/) with the IslandPath-DIOMB, SIGI-HMM, and IslandPicker methods. Prophage regions were predicted using the PHAge Search Tool (PHAST) web server (http://phast.wishartlab.com/), and CRISPR identification was performed using CRISPRFinder. The above website and software were accessed on 25 April 2023.

#### 2.9.2. Transcriptome Analysis of HF10 and Y11

The Y11 strain, which lacks antifungal activity, was generated through the chemical mutagenesis of HF10 using nitrosoguanidine (NTG) [34]. HFs 10 and 11 were inoculated into LB liquid media and incubated at 37 °C for 24 h. Subsequently, the cultures were transferred to 50 mL centrifuge tubes and centrifuged at 10,000 rpm for 10 min. The supernatant was subsequently discarded, and the cells were washed three times with PBS before precipitation. The samples were then frozen in liquid nitrogen, and the frozen samples were subsequently sent to Wuhan Genomics Technology Co., Ltd., Wuhan, China. for transcriptome analysis. The SMRT-Analysis software package smrtlink8.0 (PacBio) was used for Iso-Seq data analysis. The sequences of interest recognition (smrtlink ccs), full-length transcript identification (BGI’s house software Irs: https://github.com/zhuqianhua/lrs) (V1-xbio) (accessed on 13 October 2023) (clustering (iso-Seq3 cluster), and correction (iso-Seq3 polish) were used to obtain consistent sequences of the full-length transcripts.

Considering that there may still be redundancy in the polished transcripts, we used GMAP5 (13 October 2023) [35] to align the polished transcripts of each sample to the reference genome and then combined the alignment results of each sample. Using the official tofu_collapse (https://github.com/Magdoll/cDNA_Cupcake) (accessed on 13 October 2023) program, redundancy was removed according to the position of the transcript on the genome, and then the cuffcompare [36] program was used to compare the deredundant transcript with the reference gene to obtain a detailed classification of the transcripts.

We annotated the high-quality transcripts based on seven functional databases: hmmscan (v3.1b2) [37], which was used to annotate transcripts with the Pfam (v14.6) [38] library; Blastn (v2.2.28) [39], which was used to annotate transcripts with the NT database; and Blastx (v2.2.28) [39] and Diamond (v0.8.31) [40], which were used to annotate the transcripts with the NR, KOG (v20090331) [41], KEGG (v101) [42], Swiss-Prot (release-2020_02) [43], Blast2GO (v2.5.0) [44], and NR annotation results were used for GO [45] annotation.

### 2.10. Statistical Analysis

Analysis of the variance for each index was conducted using SPSS 20.0 to compare the differences among the treatments. Prism [46] was used to assess the significance of the differences between the data. All of the analyses were based on three independent replicates, unless specified otherwise.

## 3. Results

### 3.1. Isolation and Screening of the Antagonistic Bacterium Bacillus cereus HF10

**Figure 1:**
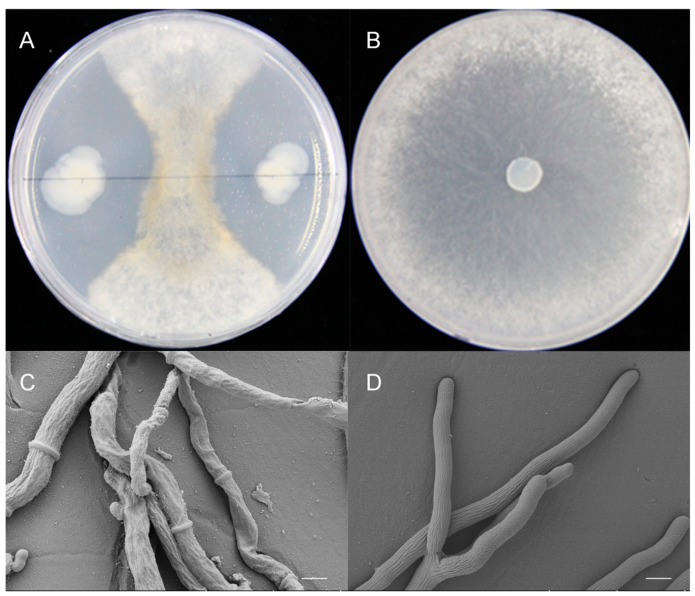
Formalization plates for the antagonistic effect of the *Bacillus cereus* HF10 strain on *S. sclerotiorum.* (**A**) HF10 (the left and right colonies) and *S. sclerotiorum* (middle sector); (**B**) *S. sclerotiorum*; (**C**) the abnormal hyphae of *S. sclerotiorum* that was inhibited by HF10, which was observed via cryo-electron microscopy; and (**D**) the normal hyphae of *S. sclerotiorum*. Bar = 100 μm.

**Figure 2:**
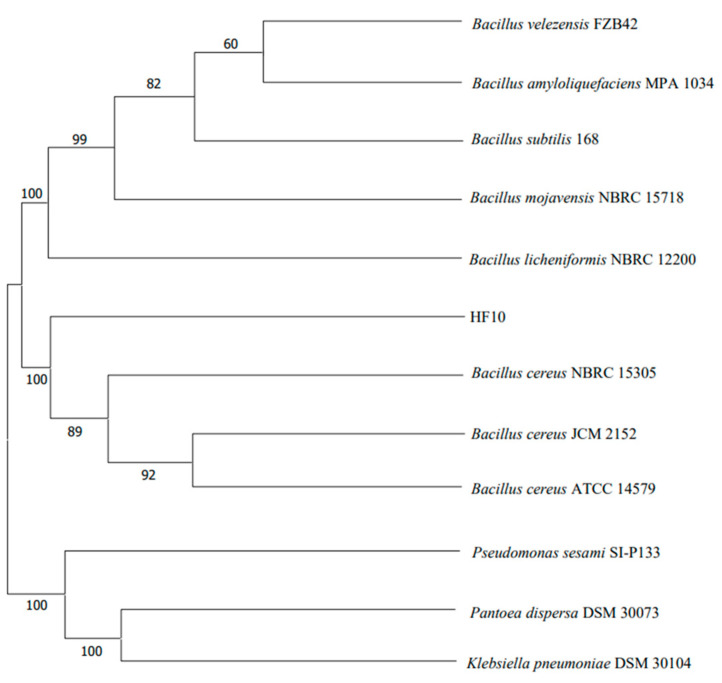
The phylogenetic tree of the HF10 strain based on 16S rDNA gene sequences. HF10 and the *B. cereus* NBRC15305, *B. cereus* JCM2152, and *B. cereus* ATCC14579 strains were clustered into a cluster with a bootstrap value of 100%. The 16S rDNA sequences were downloaded from the National Center for Biotechnology Information.

A total of 550 strains of bacteria were isolated and purified from the rhizosphere soil of the reed plants on the seaside of Yagzhou Bay, Sanya city, Hainan Province, China. One strain, named HF10, which responded with a strong antagonistic effect on *S. sclerotiorum*, was screened by a plate confrontation test and was found to have a 79% inhibitory effect (Figure 1A,B). The 16S rDNA sequence suggested that HF10 is a strain of *B. cereus* (Figure 2). In the confrontation culture, the growth of the *S. sclerotiorum* hyphae close to *B. cereus* HF10 was significantly inhibited, i.e., the color turned yellow, and the growth of mycelia was curved. The use of freezing scanning electron microscopy (FSEM) revealed that the mycelia of the normal *S. sclerotiorum* was uniform in thickness and smooth, while the mycelia of the inhibited *S. sclerotiorum* exhibited an irregular network, uneven thickness, atrophy, severe distortion, and thinning (Figure 1C,D). The mycelia growth rate method was used to test the effect of HF10 on the growth of *S. sclerotiorum* using the fermentation broth supernatant of HF10. The antagonistic effect decreased with increasing dilution, and the inhibition rates were 58%, 47%, and 30% at 10×, 20×, and 50× dilutions, respectively (Supplementary Figure S1).

### 3.2. Identification of Antagonistic Strains

#### 3.2.1. Physiological and Biochemical Characteristics

The physiological and biochemical characteristics of *B. cereus* HF10 were analyzed (Supplementary Table S1). The single-cell colony morphology and Gram staining of *B. cereus* HF10 were observed (Supplementary Figure S2). A sugar alcohol fermentation test revealed that HF10 can utilize glucose, maltose, and sucrose but not mannitol, sorbitol, lactose, or galactose. Positive results were obtained via the starch hydrolysis test, catalase test, gelatine liquefaction test, nitrate reduction test, anaerobic test, 7% NaCl test, V-P test, and oxidase test, thereby suggesting that HF10 can hydrolyze starch, produce propylene glycol, decompose gelatin, and induce antioxidant activity, as well as possess oxidases and nitrate reductase. However, a negative result was shown in the propionate test.

#### 3.2.2. Phylogenetic Tree

Based on the 16S rDNA sequence, the HF10 strain exhibited more than a 99% identity with *B. cereus*. A phylogenetic tree was constructed using MEGA-X software (10.2.4) (Figure 2), in which it was revealed that HF10 clustered with other *B. cereus* strains on the same branch of the tree. Concurrently, based on the morphological characteristics, physiological and biochemical tests, and 16S rDNA molecular identification, the strain was conclusively identified as *B. cereus*.

### 3.3. Determination of the Antifungal Activity Spectrum of the HF10 Strain

**Figure 3:**
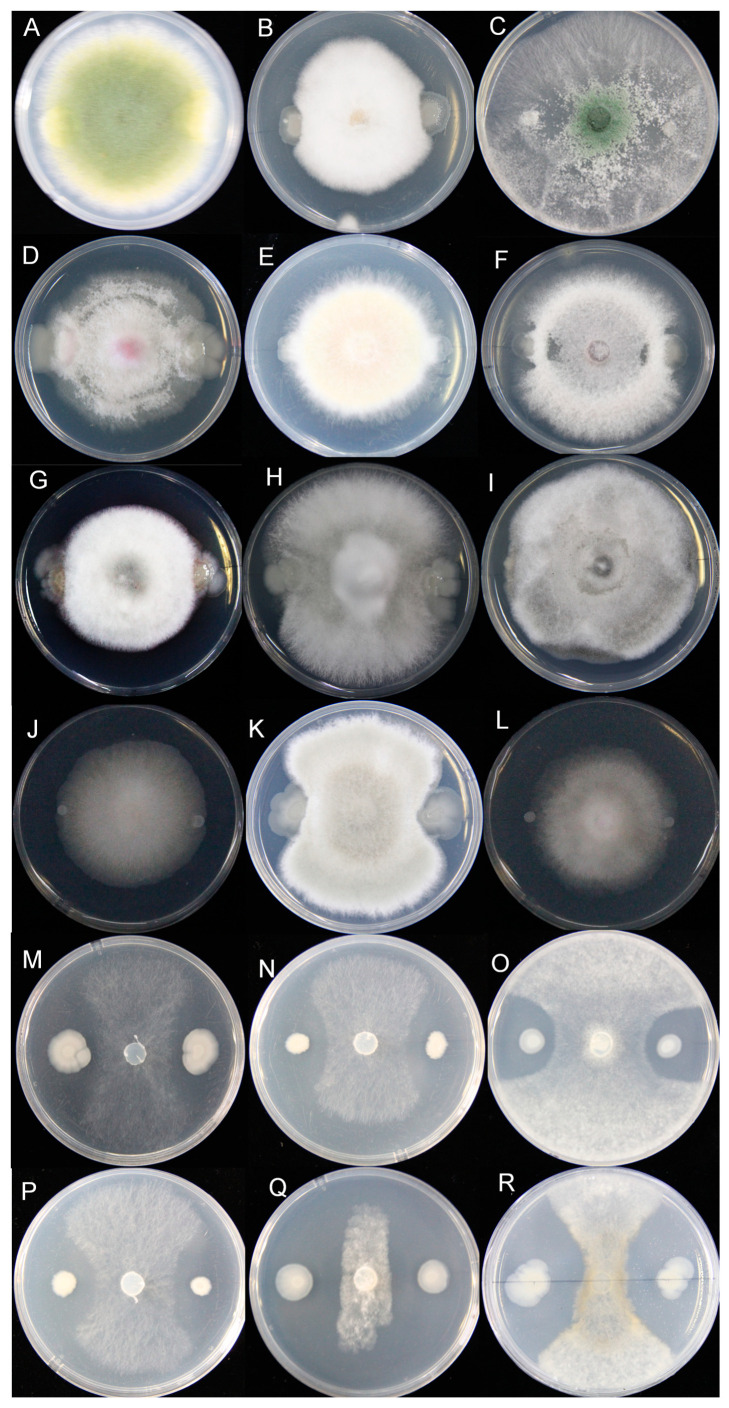
Determination of the antifungal activity and effect spectrum of HF10. (**A**–**L**), *Aspergillus flavus*, *Fusarium equiseti*, *Trichoderma asperellum*, *Fusarium oxysporum*, *Fusarium graminearum*, *Fusarium verticillioides*, *Corynespora cassiicola*, *Colletotrichum horii*, *Alternaria gossypina*, *Cladosporium cladosporioides*, *Glomerella ciningulaia*, and *Mucor irregularis*, respectively. (**M**–**R**), *S. sclerotiorum* WH13, *S. sclerotiorum* 1980, *S. sclerotiorum* P134, *S. sclerotiorum* Ep-1pn A367, *S. minor*, and *S. sclerotiorum* H1, respectively.

The confrontation test results showed that the HF10 strain responded with strong antifungal activity against the fungi of *Sclerotinia,* including *S. sclerotiorum* WH13, *S. sclerotiorum* 1980, *S. sclerotiorum* P134, *S. sclerotiorum* Ep-1pn A367, *S. minor*, and *S. sclerotiorum* H1. However, there was no antifungal activity against other fungi, including *Aspergillus flavus*, *Fusarium equiseti*, *Trichoderma asperellum*, *Fusarium oxysporum*, *Fusarium graminearum*, *Fusarium verticillioides*, *Corynespora cassiicola*, *Colletotrichum horii*, *Alternaria gossypina*, *Cladosporium cladosporioides*, *Glomerella ciningulaia*, and *Mucor irregularis*. Notably, antagonistic activity specific to the pathogenic fungi of the genus *Sclerotinia* was not reported (Figure 3).

### 3.4. The HF10 Strain Strongly Inhibited S. sclerotiorum Infection in Oilseed Rape

**Figure 4:**
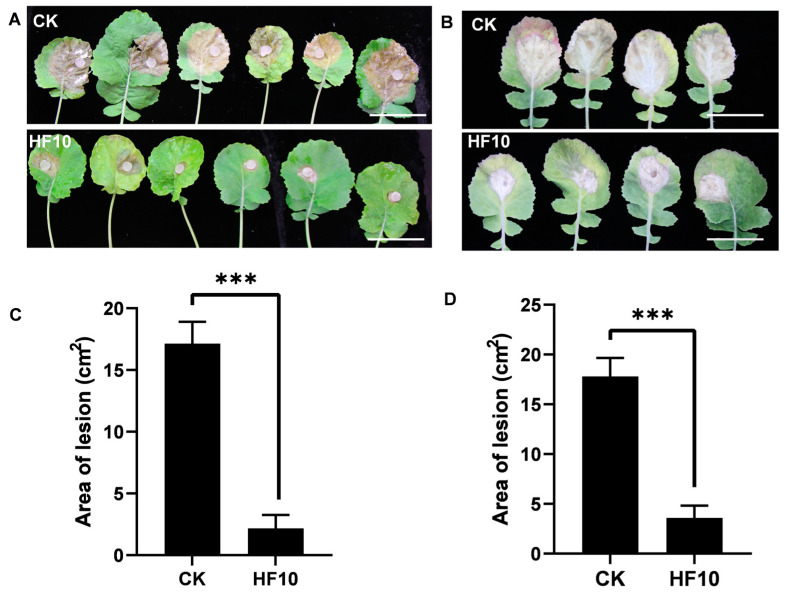
Protective effect of the HF10 strain on oilseed rape. (**A**,**B**) The phenotype of *S. sclerotiorum* infection on leaves treated with and without HF10 in vitro and in vivo, respectively. (**C**,**D**) The evaluation data of (**A**,**B**), respectively. *** *p* < 0.01 and bar = 7 cm.

The inhibitory effect of *B. cereus* HF10 on the *S. sclerotiorum* infection of oilseed rape plants was tested in vitro and in vivo. The inhibition rate of the HF10 strain on the *S. sclerotiorum* infection of oilseed rape leaves reached 82.3% according to the in vitro test (Figure 4). In vivo experiments showed that HF10 can significantly decrease the disease incidence of oilseed rape sclerotinia (Figure 4). Oilseed rape leaves and plants treated with *B. cereus* HF10 exhibited less disease and normal growth, while untreated plants displayed typical symptoms of Sclerotinia. In the in vivo experiment, the disease index of CK was 56.66 ± 3.33, while the disease index of the HF10 treatment was 16.04 ± 1.23. Therefore, the biocontrol efficacy of the HF10 strain was 71.7%. These findings suggest that *B. cereus* HF10 could be potentially applied as a biocontrol agent for managing Sclerotinia in oilseed rape.

### 3.5. The Colonization of Oilseed Rape by the HF10 Strain

Seven days after inoculation of the HF10 strain, the colonies on the leaf surface were cultured on a solid plate media, which was supplemented with spectinomycin, and the colonization amount reached 1.2 × 10^6^ cfu/g, thereby indicating that the HF10 strain could colonize the leaf surface of oilseed rape well and can possess a strong colonization ability.

### 3.6. Investigation of the Antibacterial Substances of the HF10 Strain

**Figure 5:**
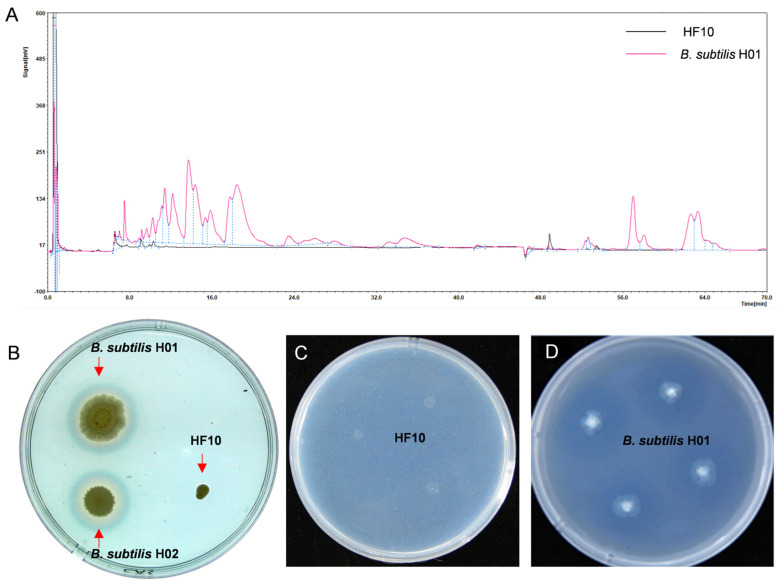
Detection of the HF10 antifungal substance. (**A**) The black line represents HF10, while the purple line corresponds to the *B. subtilis* H01 secreting lipopeptides, fengycin at 7–28 min, and surfactin at 50–67 min, as detected by HPLC. (**B**) The two colonies on the left are *B. subtilis* H01 and *B. subtilis* H02, which produce siderophores. The yellow circle around their colonies indicates that the colony on the right is HF10, and there is no yellow circle around the colony (**C**,**D**). and (**C**) show HF10, and (**D**) is a positive control *B. subtilis* H01 that produced β-1,3 glucanase and is shown as a transparent circle around the colony.

The lipopeptides secreted from *Bacillus* species demonstrated a strong antifungal activity [47]. To test whether HF10 can secrete lipopeptides, we extracted and detected the lipopeptides. We further detected other antifungal substances, such as siderophores and β-1,3 glucanase, and the results showed that—compared with the positive controls *B. subtilis* H01 and *B. subtilis* H02 (which produce the lipopeptides of surfactin and fengycin), as well as the siderophores and β-1,3 glucanase—HF10 did not produce surfactin or fengycin (Figure 5A), nor siderophores (Figure 5B) or β-1,3 glucanase (Figure 5C,D).

### 3.7. Genome Assembly of HF10 De Novo

#### 3.7.1. Genomic Features of the HF10 Strain

**Figure 6:**
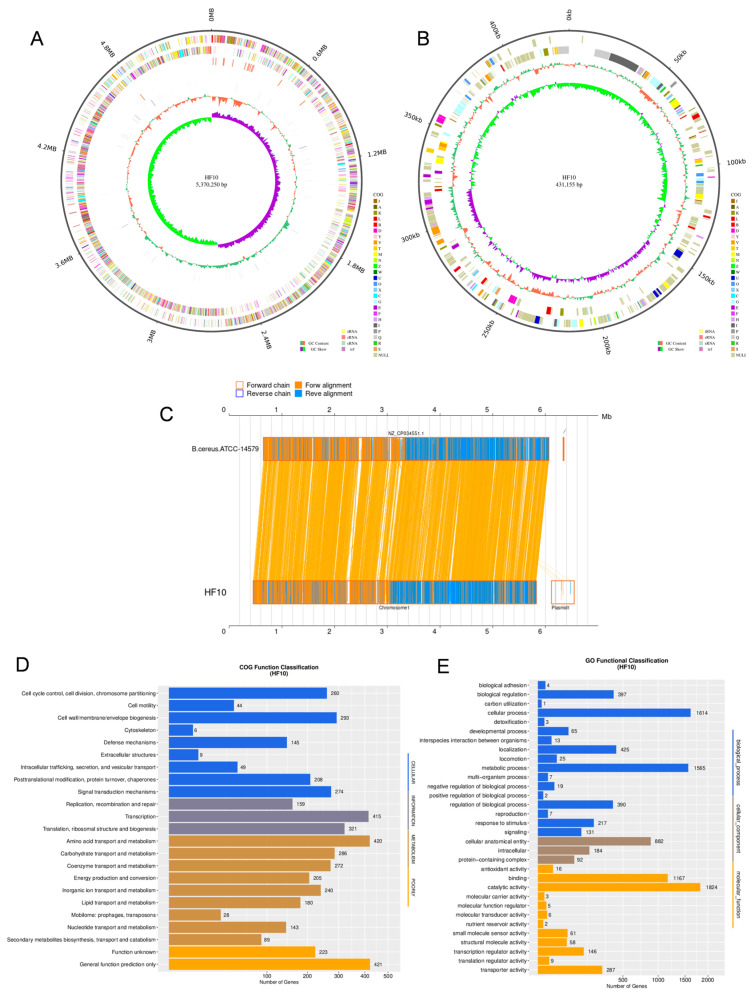
Genomic features of the HF10 strain. (**A**,**B**) Circular representation of the *B. cereus* HF10 genome. The genome size was established from the outer to the inner genome. Forward-strand genes are colored according to the cluster of the orthologous group (COG) classification, the reverse-strand genes are colored according to the cluster of the orthologous group (COG) classification and GC content (%), and the inner ring depicts the GC skew. (**C**) Nucleic acid collinearity analysis of the representative *B. cereus* HF10 and *B. cereus* ATCC-14597 strains. (**D**) COG functional classification of the protein-encoding genes of *B. cereus* HF10. (**E**) Gene ontology (GO) functional categories of the protein-encoding genes in *B. cereus* HF10.

The complete genome sequence of *B. cereus* HF10 was assembled; it contained one chromosome that was 5,370,250 bp long and one plasmid that was 431,155 bp long (Figure 6A,B). The average GC content was 35.25% on the chromosome and 32.31% on the plasmid. ANI analysis revealed that HF10 shared an average ANI value of 98% with other *B. cereus* strains, and it was highly homologous to the ATCC-14597 strain (ANI value > 98%) (Figure 6C). A total of 5786 protein-coding genes, 42 rRNAs, and 108 tRNAs were detected on the chromosome, and 368 protein-coding genes were detected in the plasmid. The whole-genome sequence and plasmid sequence used were registered at the BIG Submission (https://ngdc.cncb.ac.cn/gsub/) (accessed on 21 January 2024), and the serial number was PRJCA022776.

Most of the proteins with known functions were associated with amino acid transport and metabolism; transcription; carbohydrate transport and metabolism; inorganic ion transport and metabolism; and the cell wall, membrane, and envelope biogenesis (Figure 6D). GO annotation revealed that most of these genes were assigned to the functional categories of metabolic processes, cellular processes, catalytic activity, and binding (Figure 6E). These functional categories indicated that this bacterial strain has a strong metabolic capacity. Further annotation via antiSMASH revealed that the HF10 strain might harbor multiple gene clusters that encode petrobactin, bacillibactin, fengycin, and molybdenum cofactors (Table 1). Most of the functional genes and all of the gene clusters were located on the chromosome, and the plasmid largely comprised genes with unknown functions.

#### 3.7.2. Transcriptome Results for the HF10 and Y11 Strains

**Figure 7:**
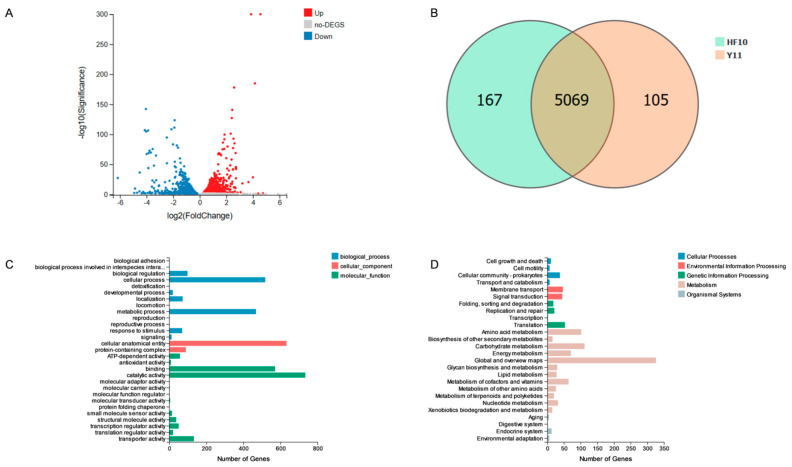
Transcriptomic analysis of HF10 and Y11. (**A**) Volcano plot illustrating the genes differentially expressed between HF10 and Y11 at a significance level of *p* < 0.05 and log2(FC). (**B**) Venn diagram depicting the genes with differential expressions between HF10 and Y11 (*p* < 0.05); notably, 167 genes were exclusively expressed in HF10. (**C**) GO enrichment analysis of DEGs at a significance level of *p* < 0.05. (**D**) KEGG pathway enrichment analysis (*p* < 0.05).

Y11 is a strain that is obtained by the NTG mutagenesis of HF10 and lacks antifungal function. Resequencing revealed that 103 genes at Y11 were mutated, namely 75 missense mutations, 14 nonsense mutations, and 14 synonymous mutations. Transcriptome analysis revealed that, when compared with HF10, Y11 exhibited an increase in the expression of 746 genes (Figure 7A) and a reduction in the expression of 708 genes. Notably, 167 genes were uniquely expressed in HF10 (Figure 7B). Among the genes exhibiting decreased expression, 16 genes were associated with the predicted bacteriostatic substances petrobactin and bacillibactin. The genes included *asbA*, *asbB*, *asbC*, *asbD*, *asbE*, and *asbF*. For bacillibactin, the associated genes were *entA*, *entB*, *entC*, *entE*, *dhbB*, *dhbE*, *vibB*, *vibE*, *mxcE*, and *mxcF*.

GO enrichment analysis revealed that the genes upregulated in HF10, when compared with Y11, were distributed across the biological process, cellular component, and molecular function categories. Among the biological processes, 1261 genes were identified and were found to be involved in cellular processes and single-organism processes. Among the cellular components, 723 genes were found to be mainly distributed in the cellular anatomical entities and protein-containing complexes. In the molecular function category, 1632 genes were identified, with predominant involvement in binding and catalytic activity processes (Figure 7C).

The enrichment analysis indicated that, when compared with Y11, HF10 exhibited a significant increase in KEGG pathway enrichment, with 1389 pathways identified. These pathways were found to be predominantly involved in metabolism, whereby they occurred in 1110 pathways that were associated with changes and increases (Figure 7D). The genes mentioned above exhibited enrichment in the Global and Overview Maps, as well as in the Metabolism of Terpenoids and Polyketides pathways.

## 4. Discussion

Oilseed rape is an important cash crop in China, Canada, and Australia [2] because it contains up to 49% fat and more than 20% protein; thus, there is strong interest in oilseed rape worldwide. Crop damage caused by plant diseases is the main threat to rural family income and global food security [48]. The oilseed rape Sclerotinia disease caused by the soil and airborne fungus *S. sclerotiorum* strongly affects oilseed rape yield and oil quality [49]. Studies have shown that *S. sclerotiorum* can cause oilseed rape stem rot [50], the rapid decomposition of tissue, and rapid withering [51]. Biological control is an important measure for treating plant diseases, and such research is growing rapidly [52]. In our study, a new strain, *B. cereus* HF10, was isolated and found to have a strong antagonistic effect on *S. sclerotiorum* (Figure 1).

Many kinds of bacteria, such as those of the *Bacillus* genus and *Pseudomonas* genus, have potential for biological control, as well as a broad spectrum of antifungal effects on pathogenic fungi [53,54]. The bacteria of the *Bacillus* genus are the most commonly used for the development of commercial biological control agents because they have a high tolerance to environmental stress, as well as a high capacity for promoting plant growth and the production of antibacterial compounds [55]. However, many *Bacillus* species have antagonistic effects not only on pathogenic fungi, but also on some probiotic fungi. The *B. cereus* HF10 strain isolated in this study produced no inhibitory effect on a variety of fungi, such as *Aspergillus flavus*, *Fusarium equiseti*, *Trichoderma asperellum*, *Fusarium oxysporum*, *Fusarium graminearum*, *Fusarium verticillioides*, *Corynespora cassiicola*, *Colletotrichum horii*, *Alternaria gossypina*, *Cladosporium cladosporioides*, *Glomerella ciningulaia*, and *Mucor irregularis* (Figure 3A–L), but it did exhibit specific inhibitory activity against the fungi of the genus *Sclerotinia* (Figure 3M–R). However, the inhibitory effect of HF10 on other fungi needs to be further evaluated. We further tested its antibacterial activity against *Escherichia coli*, *Staphylococcus aureus*, and *Agrobacterium tumefaciens*, and we found that it was not antagonistic to these bacteria (Supplementary Figure S3). The specificity of the antagonistic effect of HF10 against *Sclerotinia* is rare among reported biocontrol agents, and it may have advantages for controlling *Sclerotinia* while rarely affecting other microorganisms on oilseed rape and in the environment.

In the discovery of probiotics, diverse taxonomic groups, including fungi [56], bacteria [23], actinomyces [57], and viruses [58], have been demonstrated to be effective potential biocontrol agents for managing the Sclerotinia stem rot caused by *S. sclerotiorum* [59], in which the highest control effectiveness was 61% after 7 days of in vivo inoculation [60]. In our in vivo experiment, the efficacy of *B. cereus* HF10 against Sclerotinia was 71.7% after 7 days. *B. cereus* HF10 demonstrated a promising control effect and application potential.

In previous studies, *Bacillus cereus* has been widely used in biological control and has been shown to have antagonistic effects on a variety of fungi [61]. For example, *B. cereus* YN917 for the biocontrol of rice blast and *B. cereus* YN917 for the biocontrol of rice blast antagonize pathogens by producing secondary metabolites such as phenazine and siderophores [61]. In the complete genome sequence of the YN917 strain (GenBank accession number IPRJNA687285 for the BioProject), six genes related to siderophores were found in the ABC transporter pathway. Genes related to siderophores, including *entA*, *entB*, *entC*, and *entE*, were identified, and the genes related to iron metabolism regulated by IdeR, including *nuoA*, *nuoB*, *nuoC*, *nuoD*, *nuoH*, *nuoI*, *nuoJ*, *nuoK*, *nuoL*, *nuoM*, and *nuoN*, were also identified [62]. In our study, HF10 inhibited only *Sclerotinia*, so it is important to explore its genetic basis. In the genome of HF10, four gene clusters may be involved in the biosynthesis of compounds with biocontrol properties, including petrobactin, bacillibactin, fengycin, and molybdenum cofactors. The genes related to the siderophore metabolism in HF10 cells were *entA*, *entB*, *entC*, *entE*, *dhbB*, *dhbE*, *vibB*, *vibE*, *mxcE*, and *mxcF*. There were six more related genes than *B. cereus* YN917 for the predicted siderophore production. However, in our study, siderophores were not detected in HF10.

To explore the antimicrobial effects of these substances, we generated a Y11 mutant, which showed a loss of inhibitory function against Sclerotinia. We resequenced this gene and found 72 nonsynonymous mutations, among which the genes associated with antibiotic substances included *mshD*, *speB*, *opuD*, and *betL*, which were related to mycothiol synthase, agmatinase, and the glycine betaine transporter, thereby suggesting that these genes may be related to the production of antifungal substances.

The transcriptome is an important tool for the analysis of gene functions. We further analyzed the transcriptomes of HF10 and its mutants, and we found that the genes related to polyketones, including *asbA*, *asbB*, *asbC*, *asbD*, *asbE*, *asbF*, *entA*, *entB*, *entC*, *entE*, *dhbB*, *dhbE*, *vibB*, *vibE*, *mxcE*, and *mxcF*, were downregulated in bacillibactin. However, whether these changes are related to the production of antifungal substances needs to be further investigated.

In our study, we detected the antifungal substances of HF10 and found that surfactin, fengycin, siderophores, and β-1,3-glucanase were not produced. The genome sequence data and the transcriptome analysis data of HF10 and its mutants could provide valuable clues for further research on the identification of specific antifungals of HF10.

Although we compared the genome and transcriptome of HF10 and the mutant Y11, we still could not obtain more precise clues to acquire antifungal metabolites specific to *Sclerotinia*. Based on the characteristics of HF10, we speculated that previously reported conventional broad-spectrum antifungal substances could not be the metabolites secreted by HF10. Instead, some of the molecules specific to *Sclerotinia* may be considered, such as small RNAs, specific short peptides, etc., (as per the personal communication with Professor Gang Liu and Professor Weishan Wang, Institute of Microbiology, CAS). The antifungal mechanisms of HF10 that are specific to *Sclerotinia* are worthy of exploring with considerable efforts.

In conclusion, a novel strain of *B. cereus*, HF10, was isolated. It has a strong antagonistic effect that is specific to *Sclerotinia* pathogens and could be used for the development of an effective biological control agent against Sclerotinia in oilseed rape.

## Figures and Tables

**Table 1:** Prediction of the bacteriostatic substance-related genes in the HF10 strain.

Gene Cluster	Pathway	Function	Identity (%)
Petrobactin	Other	Ironophore; accumulates and takes up iron ions	100%
Bacillibactin	NRP	Accumulates and takes up iron ions	85%
Fengycin	NRP	Anti-fungal; signal for plant cell	40%
Molybdenum cofactor	Other	Nitrogen metabolism; sulfur metabolism	17%

## Data Availability

Data are contained within the article. The genome sequence data of HF10 is available.

## Supplementary Materials

The following supporting information can be downloaded: https://www.mdpi.com/article/10.3390/microorganisms12030611/s1, Table S1: Identification of the physiological and biochemical properties of HF10; Figure S1: Inhibitory effect of the sterile supernatant from fermentation with HF10 on *Sclerotinia sclerotiorum*; Figure S2: Colony and cell morphology of *B. cereus* HF10; Figure S3: The HF10 strain that had no inhibitory effect on bacteria.
