# Supplementary material for: A Novel Strain of Bacillus cereus with a Strong Antagonistic Effect Specific to Sclerotinia and Its Genomic and Transcriptomic Analysis
Source: Microorganisms. 2024 Mar 19;12(3):611. doi: 10.3390/microorganisms12030611 (PMC10974711; doi:10.3390/microorganisms12030611)
Supplement: Supplementary file 1 [file microorganisms-12-00611-s001.zip › microorganisms-2875709-supplementary.pdf]

A Novel Strain of *Bacillus cereus* with a Strong Antagonistic Effect Specific to *Sclerotinia* and Its Genomic and Transcriptomic Analysis

Wanfu Ma [1,2,†], Jinhao Ding [2,3,†], Qingyun Jia [2,3], Qianru Li [2,3], Shanhai Jiao [4], Xupeng Guo [2], Chengming Fan [2,*], Yuhong Chen [2,*] and Zanmin Hu [2,3]

[1] College of Tropical Agriculture and Forestry, Hainan University, Haikou 570228, China; mawanfu1234@163.com
[2] Key Laboratory of Seed Innovation, Institute of Genetics and Developmental Biology, Innovation Academy for Seed Design, Chinese Academy of Sciences, Beijing 100101, China; jhding@genetics.ac.cn (J.D.); qyjia@genetics.ac.cn (Q.J.); qrli@genetics.ac.cn (Q.L.); guoxvpeng@163.com (X.G.); zmhu@genetics.ac.cn (Z.H.)
[3] College of Advanced Agricultural Sciences, University of Chinese Academy of Sciences, Beijing 100049, China
[4] AUSCA Oils and Grains Industries Co., Ltd., Fangchenggang 538000, China; jsh123456@126.com
* Correspondence: cmfan@genetics.ac.cn (C.F.); yhchen@genetics.ac.cn (Y.C.); Tel./Fax: +86-010-64807626
† These authors contributed equally to this work.

Table S1: Identification of the physiological and biochemical properties of HF10.

Physiological and biochemical tests	results	Physiological and biochemical tests	results
Glucose fermentation test	+	Catalase test	+
Maltose fermentation test	+	Gelatine liquefaction test	+
Sucrose fermentation test	+	Nitrate reduction test	+
Mannitol fermentation test	-	Anaerobic test	+
Sorbitol fermentation test	-	7% NaCl (sodium chloride) test	+
Lactose fermentation test	-	Voges-Proskauer test	+
Galactose fermentation test	-	Oxidase test	+
Sodium propionate test	-	Starch hydrolysis test	+

Note: "+" represents a positive reaction, and "-" represents a negative reaction.

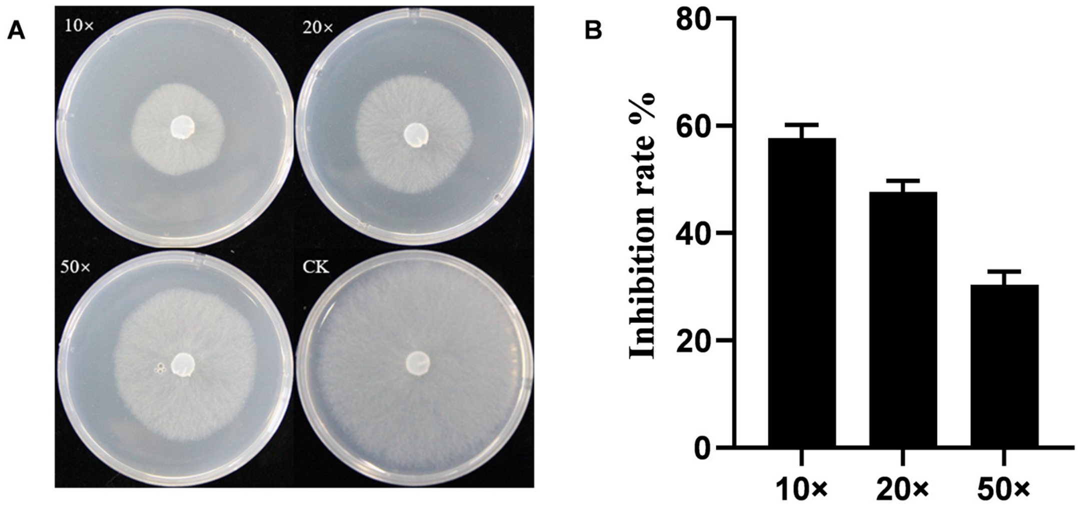

Figure S1: Inhibitory effect of sterile supernatant from fermentation with HF10 on Sclerotinia sclerotiorum. Inhibitory effect of sterile supernatant from fermentation with HF10 broth diluted to 10×, 20×, and 50× on Sclerotinia sclerotiorum. As the dilution time increased, the inhibitory effect decreased (A), with inhibition rates of 58%, 47%, and 30% at 10×, 20×, and 50×, respectively (B).

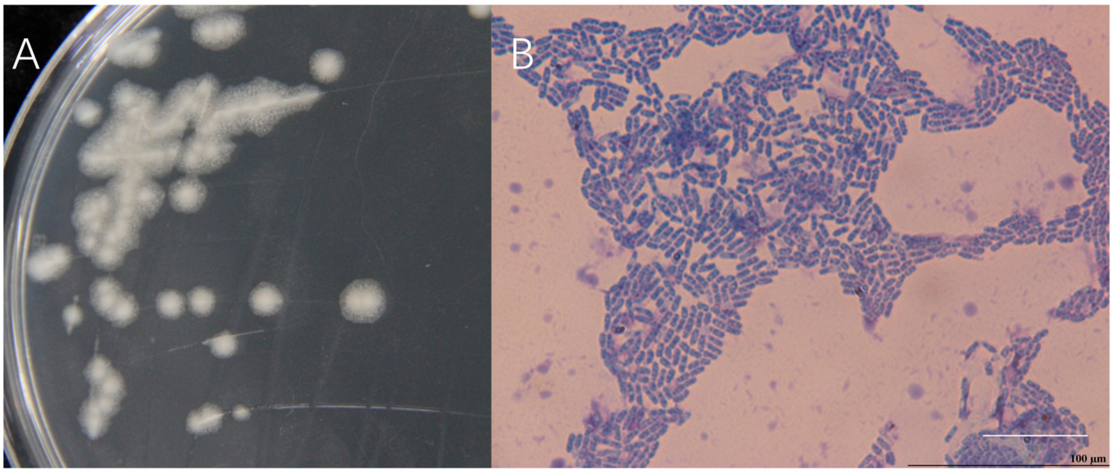

Figure S2: Colony and cell morphology of *B. cereus* HF10. A, Colony of strain HF10; white, flat, and wet opaque surface; suborbicular shape. B, Cells stained with 0.1% crystal violet stain solution, indicating a rod-shaped structure. Bar = 50 μm.

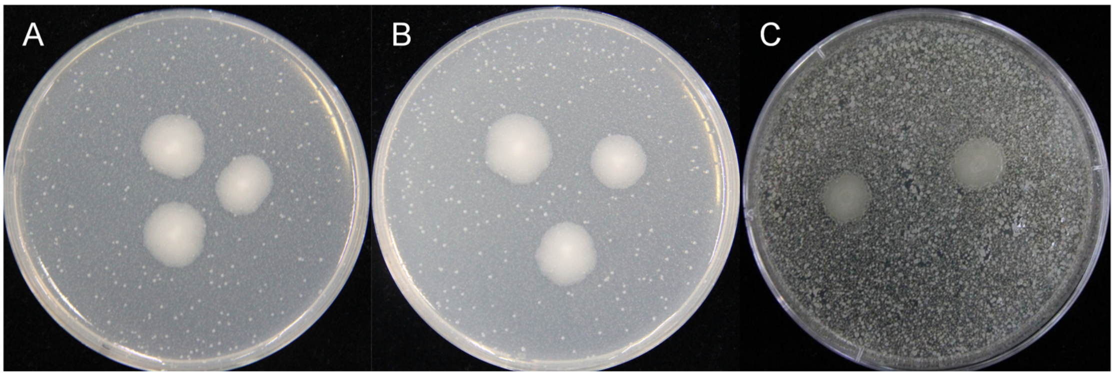

Supplementary Figure S3: HF10 had no inhibitory effect on bacteria**.** We tested its antibacterial activity against *Escherichia coli* (A), *Staphylococcus aureus* (B), and *Agrobacterium tumefaciens* (C) and found that it had no inhibitory effect on these bacteria.

>16S rDNA sequencing results of HF10

AGGGGCCCGTGGCCGGGCTGCTATACATGCAGTCGAGCGAATG

GATTAAGAGCTTGCTCTTATGAAGTTAGCGGCGGACGGGTGAGT

AACACGTGGGTAACCTGCCCATAAGACTGGGATAACTCCGGGA

AACCGGGGCTAATACCGGATAACATTTTGAACCGCATGGTTCGA

AATTGAAAGGCGGCTTCGGCTGTCACTTATGGATGGACCCGCGT

CGCATTAGCTAGTTGGTGAGGTAACGGCTCACCAAGGCAACGA

TGCGTAGCCGACCTGAGAGGGTGATCGGCCACACTGGGACTGA

GACACGGCCCAGACTCCTACGGGAGGCAGCAGTAGGGAATCTT

CCGCAATGGACGAAAGTCTGACGGAGCAACGCCGCGTGAGTG

ATGAAGGCTTTCGGGTCGTAAAACTCTGTTGTTAGGGAAGAAC

AAGTGCTAGTTGAATAAGCTGGCACCTTGACGGTACCTAACCA

GAAAGCCACGGCTAACTACGTGCCAGCAGCCGCGGTAATACGT

AGGTGGCAAGCGTTATCCGGAATTATTGGGCGTAAAGCGCGCG

CAGGTGGTTTCTTAAGTCTGATGTGAAAGCCCACGGCTCAACC

GTGGAGGGTCATTGGAAACTGGGAGACTTGAGTGCAGAAGAG

GAAAGTGGAATTCCATGTGTAGCGGTGAAATGCGTAGAGATATG

GAGGAACACCAGTGGCGAAGGCGACTTTCTGGTCTGTAACTGA

CACTGAGGCGCGAAAGCGTGGGGAGCAAACAGGATTAGATACC

CTGGTAGTCCACGCCGTAAACGATGAGTGCTAAGTGTTAGAGG

GTTTCCGCCCTTTAGTGCTGAAGTTAACGCATTAAGCACTCCGC

CTGGGGAGTACGGCCGCAAGGCTGAAACTCAAAGGAATTGAC

GGGGGCCCGCACAAGCGGTGGAGCATGTGGTTTAATTCGAAGC

AACGCGAAGAACCTTACCAGGTCTTGACATCCTCTGAAAACCC

TAGAGATAGGGCTTCTCCTTCGGGAGCAGAGTGACAGGTGGTG

CATGGTTGTCGTCAGCTCGTGTCGTGAGATGTTGGGTTAAGTCC

CGCAACGAGCGCAACCCTTGATCTTAGTTGCCATCATTAAGTTG

GGCACTCTAAGGTGACTGCCGGTGACAAACCGGAGGAAGGTG

GGGATGACGTCAAATCATCATGCCCCTTATGACCTGGGCTACAC

ACGTGCTACAATGGACGGTACAAAGAGCTGCAAGACCGCGAG

GTGGAGCTAATCTCATAAAACCGTTCTCAGTTCGGATTGTAGGC

TGCAACTCGCCTACATGAAGCTGGAATCGCTAGTAATCGCGGAT

CAGCATGCCGCGGTGAATACGTTCCCGGGCCTTGTACACACCGC

CCGTCACACCACGAGAGTTTGTAACACCCGAAGTCGGTGGGGT

AACCTTTTTGGAGCCAGCCGCCTAAGTGAACAGAGG
